# Revealing Less Derived Nature of Cartilaginous Fish Genomes with Their Evolutionary Time Scale Inferred with Nuclear Genes

**DOI:** 10.1371/journal.pone.0066400

**Published:** 2013-06-25

**Authors:** Adina J. Renz, Axel Meyer, Shigehiro Kuraku

**Affiliations:** Chair in Zoology and Evolutionary Biology, Department of Biology, University of Konstanz, Konstanz, Germany; Laboratoire Arago, France

## Abstract

Cartilaginous fishes, divided into Holocephali (chimaeras) and Elasmoblanchii (sharks, rays and skates), occupy a key phylogenetic position among extant vertebrates in reconstructing their evolutionary processes. Their accurate evolutionary time scale is indispensable for better understanding of the relationship between phenotypic and molecular evolution of cartilaginous fishes. However, our current knowledge on the time scale of cartilaginous fish evolution largely relies on estimates using mitochondrial DNA sequences. In this study, making the best use of the still partial, but large-scale sequencing data of cartilaginous fish species, we estimate the divergence times between the major cartilaginous fish lineages employing nuclear genes. By rigorous orthology assessment based on available genomic and transcriptomic sequence resources for cartilaginous fishes, we selected 20 protein-coding genes in the nuclear genome, spanning 2973 amino acid residues. Our analysis based on the Bayesian inference resulted in the mean divergence time of 421 Ma, the late Silurian, for the Holocephali-Elasmobranchii split, and 306 Ma, the late Carboniferous, for the split between sharks and rays/skates. By applying these results and other documented divergence times, we measured the relative evolutionary rate of the Hox A cluster sequences in the cartilaginous fish lineages, which resulted in a lower substitution rate with a factor of at least 2.4 in comparison to tetrapod lineages. The obtained time scale enables mapping phenotypic and molecular changes in a quantitative framework. It is of great interest to corroborate the less derived nature of cartilaginous fish at the molecular level as a genome-wide phenomenon.

## Introduction

**Figure 1:**
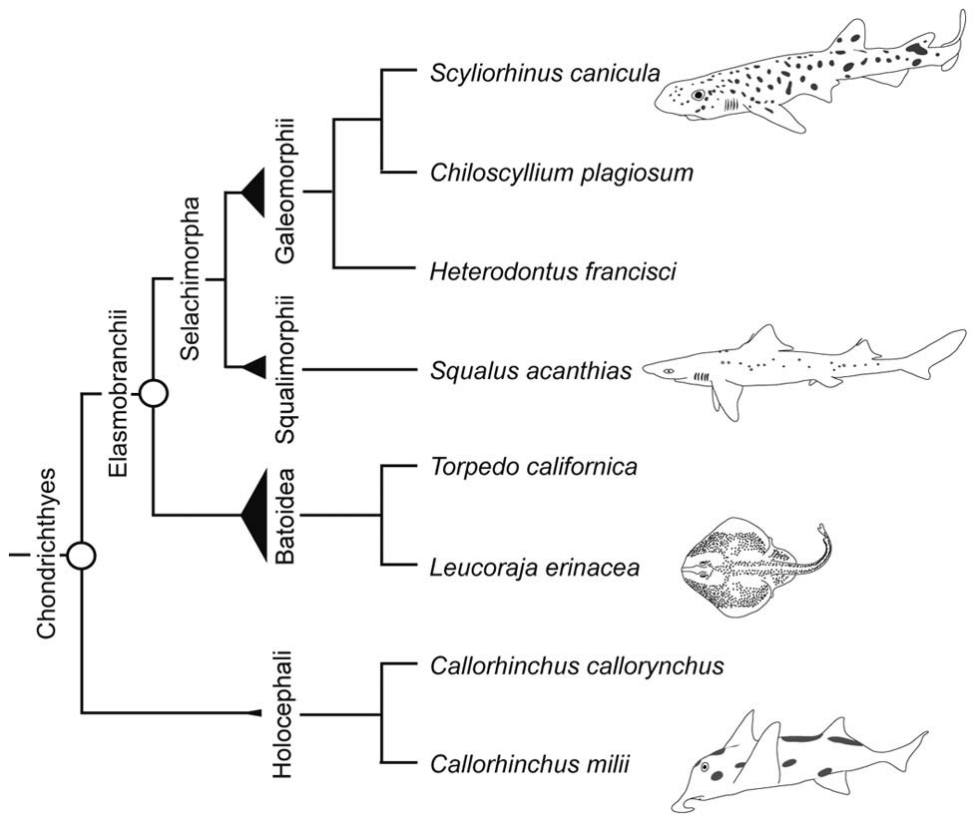
Relationship of chondrichthyan species. Species tree illustrating the relationship of all chondrichthyan species employed in our analyses, either in the divergence time study or evolutionary rate analysis (see text for alternative views of the phylogenetic relationship). Circles indicate the nodes referred to in the divergence time analysis. Widths of triangles are proportional to the numbers of species for individual groups according to Compagno et al. [69].

Chondrichthyans (cartilaginous fishes) occupy a key phylogenetic position among extant vertebrates as one of the early-branching lineages [1]–[3]. Their fossils are considered to be well preserved, largely because of the abundant deposits of dental material [4]. Living chondrichthyans are divided into two subclasses, Holocephali (chimaeras) and Elasmobranchii (sharks, rays and skates) (Figure 1). Based on the fossil records, Holocephali and Elasmobranchii are estimated to have diverged in the lowermost Devonian 410 million years before present (Ma) [5]. The monophyly of each of the two groups was strongly supported by morphological analyses [4], [6], [7], and has been reinforced by recent studies using several genes in the mitochondrial DNA (mtDNA) [8], [9]. More controversially discussed is the relationship between batoids (rays and skates) and sharks inside Elasmobranchii. Earliest morphological studies supported the basal dichotomy between sharks and batoids [10], [11] (Figure 1). However, subsequent extensive analyses of morphological variation suggested that batoids were descendants of one derived shark lineage, which is called the “Hypnosqualea hypothesis” [12], [13]. More recently, the basal dichotomy between batoids and sharks and monophylies of each of these two groups (Figure 1) have been supported by several molecular phylogenetic studies employing genes in mtDNA [9], [14]–[17] and the nucleus-encoded *recombination activating gene 1* (*RAG1*) [18]. Based on this phylogenetic relationship, the monophyletic group containing all sharks is designated ‘Selachimorpha’ (Figure 1). Recently, diverse extinct shark species, assigned to the †Synechodontiformes, were suggested to form a monophyletic group, which is sister to all living sharks [19]. This assignment of Synechodontiformes into a monophyletic group which originated at the basal position of living sharks shifts the previously assumed minimum constraint of 190 Ma as the origin of modern sharks [20] back to the Late Permian around 250 Ma [15], [19].

Some chondrichthyan species have been subjected to various molecular phylogenetic analyses, and they suggested a decreased rate of molecular evolution in the chondrichthyan lineages [21]–[25]. Martin *et* *al.* calculated the molecular evolutionary rate using two mitochondrial genes, *cytochrome b* (cytb) and *cytochrome oxidase I (COI)*, of 13 shark species and showed their slower rate compared to the mammalian lineage [21]. Later, Martin *et* *al.* reinforced that also in nuclear-encoded genes, namely *dlx*, *heat shock protein* (*HSP) 70* and *recombination activating gene* (*RAG) 1*, the rates of molecular evolution for sharks are an order of magnitude slower than those for mammals [24].

For the “Timetree of Life” [26], Heinicke *et* *al.* performed a family-level divergence time analysis for the entire Chondrichthyes [20]. They employed nucleotide sequences of the *RAG1* gene and mitochondrial 12S and 16S ribosomal RNA (rRNA) genes previously sequenced [14], [18], [27]. Especially among the major chondrichthyan lineages, the analysis employing the *RAG1* gene resulted in divergence time estimates of 100 million years older than the estimate with 12S and 16S rRNA genes [20]. Most recently, Inoue *et* *al.* inferred divergence times for fourteen divergence points at the family level of chondrichthyans, using whole mtDNA sequences [15]. Summarizing these progresses, further analyses based on the exhaustive use of latest palaeontological data employing more nuclear genes was anticipated.

Robust phylogenetic relationships and accurate divergence times are indispensable for deeper understanding of evolutionary processes. To date, unpretentious representative genes including mitochondrial genes [14], [27] and *RAG1* [18], have been frequently selected as markers in molecular phylogenetic analyses. Nowadays, increasing molecular sequence data for diverse organisms and the bioinformatic tools for handling large-scale sequence resources have enhanced the power of so-called ‘phylogenomics’. In this study, we collected nuclear protein-coding sequences and calculated divergence times of two major nodes in the chondrichthyan lineage, the Holocephali-Elasmobranchii and the Batoidea-Selachimorpha splits (Figure 1). Based on our estimates, we measured the relative evolutionary rates of chondrichthyan lineages using available Hox A cluster sequence data.

## Results

### Genomic and transcriptomic data mining

**Figure 2:**
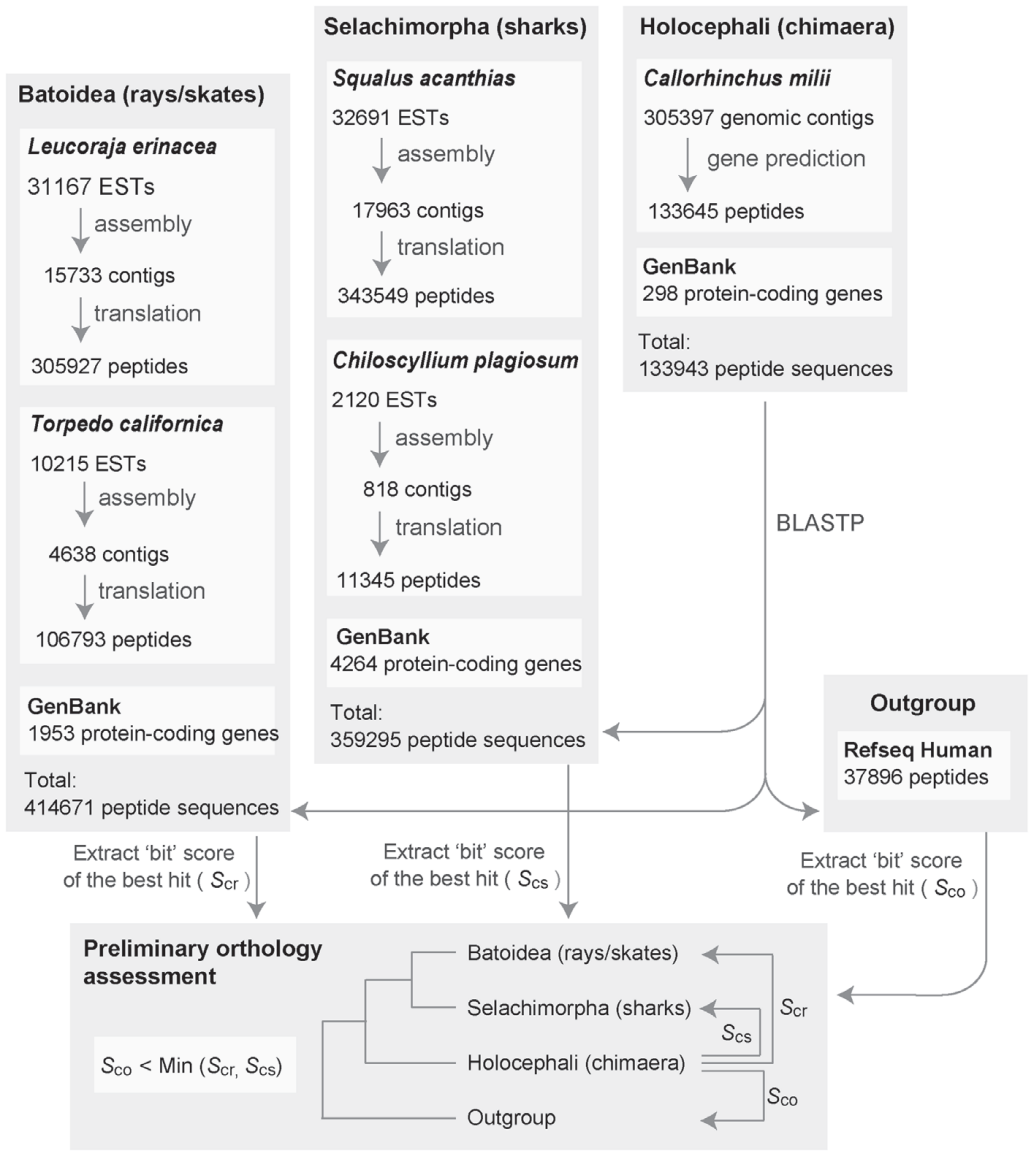
Work flow of gene family selection for divergence time estimation within chondrichthyans. See Methods for details of elasmobranch EST assembly and gene prediction on *C. milii* genomic genomic contigs. Abbreviations: EST, expressed sequence tags; GSS, genome survey sequence.

We employed still limited but fairly large-scale genomic and transcriptomic sequence resources publicly available (see Methods and Figure 2). Through the automated orthology assessment, a total of 203 candidate genes were obtained. After investigation of the corresponding alignment of each gene, 122 candidate genes were excluded from further analyses, because they yielded spurious blast hits corresponding to sequences expanded into abundant copies (>500) in the chimaera genome. These 122 discarded candidates included genes whose mammalian orthologs are known to be involved in pathogen recognition and signal transduction [e.g. the nod-like receptor (NLR) family, the tripartite motif (TRIM) family and proteins containing WD repeats or FYVE zinc-finger domains] or are predicted hypothetical genes.

**Figure 3:**
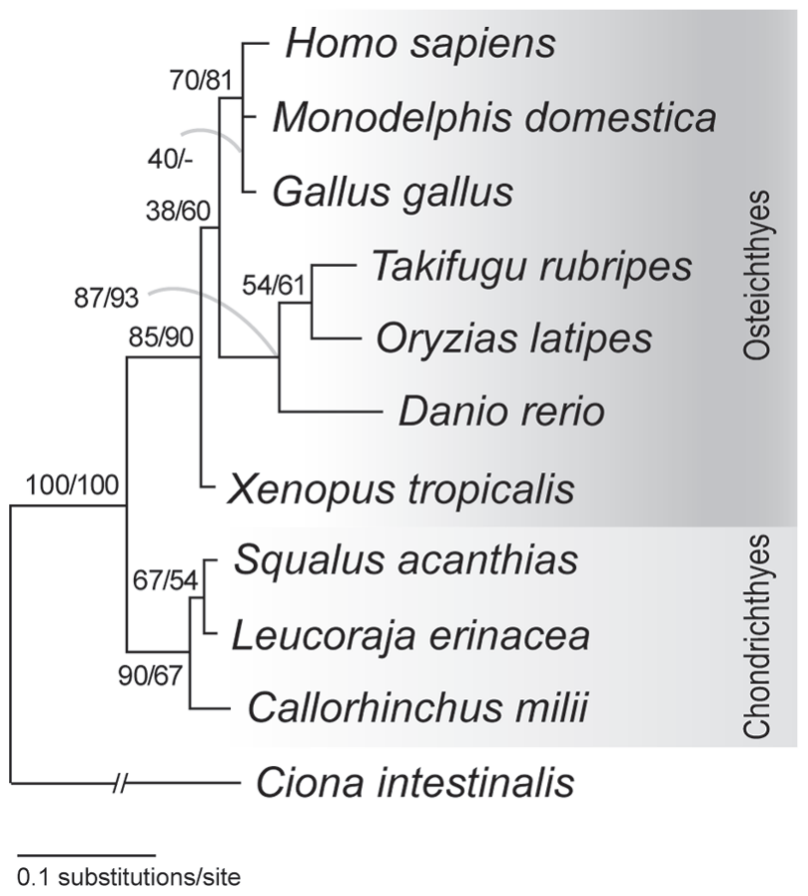
Phylogenetic tree of*RAN binding protein 1* genes. This gene is listed as candidate #9 in Table 1. The tree was reconstructed with the maximum-likelihood (ML) method (see Methods). Bootstrap values were calculated with 100 resamplings. Support values at nodes indicate, in order, probabilities in the ML and the neighbor-joining (NJ) analysis. 119 amino acid sites were included for tree inference (shape parameter for gamma distribution α = 0.38). Note that the topology of this ML tree is not consistent with the generally accepted species phylogeny, but the log-likelihood of the tree topology consistent with the species phylogeny was not significantly lower than that of the ML tree (Table 1). For this reason, this gene was included in the final dataset.

The remaining 81 candidate genes were further scrutinized regarding their phylogenetic properties, namely their orthologies within individual datasets (see Methods). Here only three candidates (#1, 13 and 18 in Table 1) yielded the maximum-likelihood (ML) tree compatible with the generally accepted species phylogeny. All other candidate genes whose phylogenetic relationship of included species differed from the generally accepted species phylogeny [1] were further analyzed with the statistical framework of the ML method. That is, we calculated the log-likelihood (log*L*) of the obtained ML tree and that of tree topology in accordance with the generally accepted species phylogeny. The example of the candidate #9, *RAN binding protein* (*RANBP*) *1* gene, is illustrated in Figure 3. In this ML tree, teleost fishes, instead of *Xenopus laevis*, are a sister group of amniotes, which does not agree with the generally accepted phylogenetic relationship [1]. However, the likelihoods of this ML tree (Figure 3) and the generally accepted species phylogeny did not significantly differ (*ΔlogL*  = 1.23±2.35).

**Table 1:** Overview of nuclear genes used for the divergence time analysis.

#	Gene name	Protein ID of human ortholog	Δlog*L* ± SE	Topology of ML tree	# of sites (aa)	Shape parameter α
1	Phosphoglycerate kinase 1 (PGK1)	NP_000282.1	ML	(((((Hs,Md),((Ol,Fr),Dr)),((Sh,Ry),Cm)),Ci),Ds);	381	0.35
2	Peptidylprolyl isomerase (cyclophilin)-like 5 isoform	NP_689542.2	4.93±7.94	((((((((Hs,Md),Gg),Xt),((Sh,Ry),Cm)),(Fr,Ol)),Dr),Ci),Dm);	225	1.30
3	DEAD (Asp-Glu-Ala-Asp) box polypeptide 20	NP_009135.3	2.84±2.98	(((((Ry,Sh),Cm),(((Hs,Md),Gg),Xt)),(((Ol,Fr),Dr),Ci)),Dm);	171	0.67
4	Sorting nexin 6 isoform b	NP_689419.2	2.22±4.89	(((((((((Hs,Md),Gg),Xt),Ol),Dr),Fr),((Cm,Ry),Sh)),Ci),Dm);	144	0.44
5	ATP/GTP binding protein-like 5 isoform 1	NP_068603.4	0.38±6.55	(((((Hs,Md),(Xt,Dr)),(Ol,Fr)),((Sh,Ry),Cm)),Ci);	157	0.43
6	Testis-specific gene A2	NP_543136.1	2.40±3.19	((((Hs,Md),Xt),(((Fr,Ol),Dr),((Ry,Sh),Cm))),Ci);	159	0.60
7	Glutamyl-prolyl tRNA synthetase	NP_004437.2	3.35±3.90	(((((((Sh,Ry),Cm),(Ol,Fr)),Xt),Gg),(Hs,Md)),Ci);	142	0.43
8	Growth arrest-specific 8	NP_001472.1	1.20±2.72	(((((((Hs,Md),Xt),Gg),Dr),((Sh,Ry),Cm)),Ci),Dm);	150	0.81
9	RAN binding protein 1	NP_002873.1	1.23±2.35	(((((Hs,(Gg,Md)),((Ol,Fr),Dr)),Xt),((Ry,Sh),Cm)),Ci);	119	0.38
10	Ring finger protein 10	NP_055683.3	0.72±1.44	(((((((Hs,Md),Gg),Xt),((Sh,Ry),Cm)),((Fr,Ol),Dr)),Ci),Dm);	118	0.95
11	MDN1, midasin homolog	NP_055426.1	3.42±3.76	((((((Hs,(Gg,Md)),(Sh,Ry)),Cm),((Ol,Fr),Dr)),Ci),Dm);	121	0.55
12	RAB, member RAS oncogene family-like 5	NP_073614.1	0.11±4.21	(((((Hs,Md),Xt),Gg),((Ol,Dr),((Sh,Ry),Cm))),Ci);	108	2.05
13	IWS1 homolog	NP_060439.1	ML	(((((((Hs,Md),Xt),Gg),(Fr,Ol)),((Cm,Sh),Ry)),Ci),Dm);	117	1.21
14	Trinucleotide repeat containing 5	NP_006577.2	1.30±2.09	((((((Hs,Md),Gg),((Ol,Fr),Dr)),Xt),((Sh,Ry),Cm)),Dm);	107	0.77
15	M-phase phosphoprotein 10	NP_005782.1	2.19±2.30	(((((((Hs,Md),Gg),Xt),(Dr,Fr)),(Sh,Ry)),Cm),Dm);	98	0.88
16	WD40 Protein Ciao 1 protein	NP_004795.1	1.26±2.04	((((((Hs,Md),(Xt,Gg)),(Fr,Dr)),((Sh,Ry),Cm)),Ci),Dm);	98	0.53
17	Ceroid-lipofuscinosis, neuronal 5 (CLN5)	NP_006484.1	0.60±1.18	((((Hs,Md),(Xt,Gg)),(Ol,Dr)),((Sh,Ry),Cm));	130	0.38
18	CCR4-NOT transcription complex	NP_055331.1	ML	(((((Hs,Md),Xt),((Fr,Ol),Dr)),((Cm,Sh),Ry)),Dm);	126	0.57
19	Splicing factor 3a, subunit 1 (SF3A1)	NP_005868.1	4.85±6.19	((((((((Hs,Md),Gg),Xt),Fr),Sh),((Cm,Ry),Ol)),Dr),Dm);	171	0.17
20	Thyroid hormone receptor interactor 12 (TRIP12)	NP_004229.1	0.63±4.44	((((Hs,(Md,Gg)),(((Ol,Fr),Dr),((Cm,Sh),Ry))),Xt),Ci);	131	0.55

The log-likelihood difference (Δlog*L*) between the ML tree and the topology based on the generally accepted species phylogeny is shown with standard error. The topology of the ML tree is shown in newick format. The number of amino acid sites used for tree inference is shown for each gene, as well as their corresponding shape parameter α for gamma distribution. Abbreviations: aa, amino acid; ML, maximum likelihood tree; Dm, *Drosophila melanogaster*; Ds, *Drosophila simulans*; Ci, *Ciona intestinalis*; Cm, chimaeras; Sh, sharks; Ry, rays/skates; Fr, *Takifugu rubripes*; Ol, *Oryzias latipes*; Dr, *Danio rerio*; Hs, *Homo sapiens*; Md, *Monodelphis domestica*; Gg, *Gallus gallus*; Xt, *Xenopus tropicalis*.

After this assessment, 20 candidate genes remained in the final dataset with each of them containing from 98 [candidate #15: *M-phase phosphoprotein* (*MPP*) *10*] to 381 [candidate #1: *phosphoglycerate kinase* (*PGK*) *1*] amino acids in the gene-by-gene alignments (Table 1). The total number of amino acids in the final concatenated dataset was 2973.

### Divergence time estimation employing nuclear protein-coding genes

#### Holocephali-Elasmobranchii split

**Figure 4:**
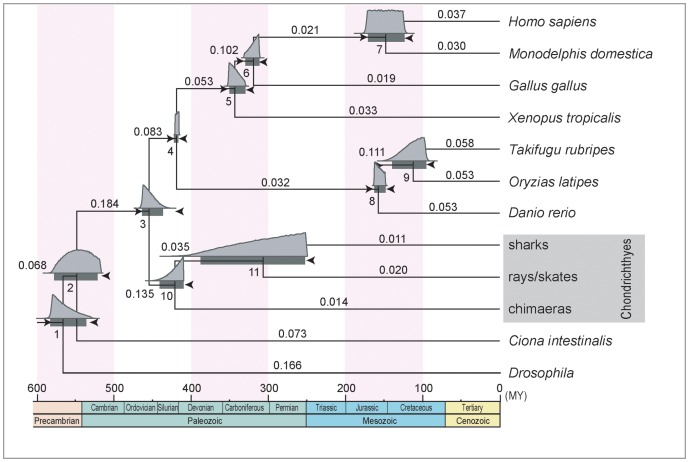
Estimated timetree of vertebrates. Timetree produced by MCMCTREE in PAML 4.4 [62] implementing the relaxed molecular clock method. A total of 19 time constraints (see Table S2) used for the calculation are shown as arrowheads at the eleven nodes. 2973 amino acid sites were analyzed derived from a total of 20 nuclear genes. Horizontal bars indicate 95% confidence intervals (CI) of the divergence time estimates. All estimates and 95% CIs are listed in Table 1. The marginal densities obtained in TRACER 1.5 are shown in light grey above the bars. Rates given by MCMCTREE are shown above the individual branches.

With the concatenated dataset, we first inferred the divergence time between Holocephali and Elasmobranchii (Figure 1). We employed fossil-based time constraints, which had two options for the split between Batoidea and Selachimorpha (node 11) (Table S2). In fact, the difference between the two options for the relatively young node did not influence the results of the time inference for the older node, namely the Holocephali-Elasmobranchii split. Our MCMCTREE analysis resulted in the estimated divergence time of about 421 Ma as posterior mean in late Silurian and a 95% confidence interval (CI) of 410–441 Ma, for the Holocephali-Elasmobranchii split (node 10 in Table 2 and Figure 4). In Figure 4, above the 95% CI time bars in the graph, the marginal densities are shown in light grey. Notably, the median divergence time (depicted above the 95% CI time bars) for node 10 is with a value of 419 Ma slightly younger than the mean value, which however aims in the direction of the previous assumed time points of this divergence [5], [15] (Figure 4).

**Table 2:** Estimated divergence times.

Node #	Constraint set I		Constraint set II	
	Time	CI	Time	CI
1	566	536–582	566	536–582
2	547	520–576	547	520–576
3	454	437–464	454	437–464
4	418	416–421	418	416–421
5	343	331–351	343	331–351
6	319	312–330	319	312–330
7	148	124–171	148	124–171
8	157	149–163	157	149–163
9	112	95–139	112	95–138
10	421	410–441	420	410–440
11	306	252–387	261	193–373

Divergence time estimates (posterior mean) and their 95% confidence intervals (CIs) in Ma (million years from present) for eleven nodes indicated in Figure 4. See Table S2 for details of the time constraints. Different minimum constraints of node #11 resulted in different divergence time estimates.

#### Batoidea-Selachimorpha split

Our second analysis target, the split between Batoidea and Selachimorpha in the Elasmobranchii lineage (node 11; also see Figure 1), was estimated at 306 Ma in the late Carboniferous period, with a 95% CI of 252–387 Ma based on constraint set I (Table 2 and Figure 4). On the other hand, based on constraint set II, this divergence was estimated at 261 Ma in the late Permian with a 95% CI of 193–373 Ma (Table 2). These 95% CIs are remarkably large presumably because of the missing constraint for any younger node. However, the estimated divergence time of 306 Ma (based on constraint set I), which is already biased through our lower constraint of 250 Ma [19], is consistent with palaeontological records. The median of the calculated marginal densities again reveals a younger divergence time of 300 Ma, which slightly aims at the direction of the previous assumed time points of divergence [15], [19].

### Molecular evolutionary rates in chondrichthyan lineages

Our application of the calculated divergence time of 306 Ma, as well as previously estimated divergence times to the Hox A cluster sequences (see Methods), revealed much lower absolute rates in the cartilaginous fish lineages in comparison to tetrapod species (Table 3). The final comparison showed an increasing evolutionary rate in Hox A genes from *H. francisci* to *X. tropicalis* (sorted from lower rate to higher rate: *Hf*, *Sc*, *Le*, *Gg*, *Hs*, *Ac, Xt*). As depicted, *H. francisci* showed the lowest evolutionary rate of the three chondrichthyans included in our study, namely 0.004×10^−8^ substitutions/site/year. *L. erinacea* as batoid representative showed the highest rate in this group with 0.009×10^−8^ substitutions/site/year, but highly similar to the rate of *S. canicula* (0.008×10^−8^ substitutions/site/year). The human Hox A genes revealed averagely an evolutionary rate of 0.028×10^−8^ substitutions/site/year. This induces a 7.0 times to 3.1 times decreased rate for chondrichthyans in comparison to human, looking at species employed in our analysis (Table 3). Considering the four tetrapod representatives, *G. gallus* showed the lowest rate of sequence divergence, however only with a 1.3 times lower rate compared to the human, and 1.4 times decreased rate in comparison to *A. carolinensis* (Table 3). By contrast with *L. erinacea*, chicken showed a 2.4 times higher evolutionary rate. This comparison, of a chondrichthyan representing the highest evolutionary rate, and a tetrapod representing the lowest evolutionary rate, reveals the largest difference between two species in our analysis, when rates are sorted according to values.

**Table 3:** Distances and evolutionary rates for chondrichthyan and tetrapod representatives.

Species pair	*Hf*, *Sc*		*Le*, *Sc*		*Hf*, *Le*		*Hs*, *Gg*		*Hs*, *Ac*		*Hs*, *Xt*	
# of aa sites	3121		2891		3128		2341		2344		2748	
Divergence time (Ma)	203		306		306		312		312		330	
Distance	*Hf*-*Cm*	0.052	*Le*-*Cm*	0.073	*Hf*-*Cm*	0.054	*Hs*-*Cm*	0.223	*Hs*-*Cm*	0.205	*Hs*-*Cm*	0.212
	*Sc*-*Cm*	0.063	*Sc*-*Cm*	0.068	*Le*-*Cm*	0.068	*Gg*-*Cm*	0.203	*Ac*-*Cm*	0.218	*Xt*-*Cm*	0.222
	*Hf*-*Sc*	0.027	*Le*-*Sc*	0.049	*Hf*-*Le*	0.038	*Hs*-*Gg*	0.155	*Hs*-*Ac*	0.183	*Hs*-*Xt*	0.200
	O-*Hf*	0.008	O-*Le*	0.027	O-*Hf*	0.012	O-*Hs*	0.087	O-*Hs*	0.085	O-*Hs*	0.095
	O-*Sc*	0.019	O-*Sc*	0.022	O-*Le*	0.027	O-*Gg*	0.068	O-*Ac*	0.098	O-*Xt*	0.105
Rate (×10^−8^)	O-*Hf*	0.004	O-*Le*	0.009	O-*Hf*	0.004	O-*Hs*	0.028	O-*Hs*	0.027	O-*Hs*	0.029
	O-*Sc*	0.009	O-*Sc*	0.007	O-*Le*	0.009	O-*Gg*	0.022	O-*Ac*	0.031	O-*Xt*	0.032

Distances (number of substitutions per site) were calculated by codeml for different pairs of species using *Callorhinchus milii* (*Cm*) as outgroup. Applying divergence times estimated in this study (306 Ma) as well as previous studies (203 Ma, 312 Ma and 330 Ma), evolutionary rates were calculated for three chondrichthyans and four tetrapod species. Abbreviations: aa, amino acid; Ma, million years ago; *Hf*, *Heterodontus francisci*; *Sc*, *Scyliorhinus canicula*; *Le*, *Leucoraja erinacea*; *Gg*, *Gallus gallus*; *Hs*, *Homo sapiens*; *Ac*, *Anolis carolinensis*; *Xt*, *Xenopus tropicalis*; O, Last common ancestor of the two selected species.

We also applied the whole Hox A cluster nucleotide sequences including non – coding regions (see Methods). This analysis showed less difference in the overall substitution rates, but the same order of species was observed, when rates are sorted according to values. *H. francisci* resulted in the lowest rate, followed by *S. canicula* and *L. erinacea*. *G. gallus*, again, showed the lowest rate of substitution in comparison between tetrapod species (data not shown).

## Discussion

### Marker gene selection and orthology assessment

More and more abundant molecular sequence data has enabled so-called ‘phylogenomics’ reconstructing more precisely the evolutionary history of many species by combining a number of independent nuclear loci ([28], [29]; also see [24] for review). The study by Li et al. on ray-finned fish phylogeny was one of the first demonstrations of *in silico* selection of phylogenetic markers [30]. This approach has been adopted in phylogenetic analyses involving a variety of taxa [31]–[34]. Similarly, in this study, we organized an *in silico* pipeline to handle available transcriptomic and genomic sequence data and chose nuclear marker genes to establish a solid time scale for cartilaginous fish evolution.

For each selected candidate gene, orthology was carefully assessed during phylogenetic analyses (see Methods). We excluded several candidates in which the tree topology based on the generally accepted phylogenetic relationships is rejected with significant statistical confidence. The 20 genes employed in our analysis were accepted in the likelihood analyses to support the currently accepted species phylogeny, although sometimes not as the ML tree (Figure 3). As divergence time estimation should be based on dataset including only orthologs, paralogy which can be detected by the inconsistency of the tree topology with the accepted species tree can mislead divergence time estimate. In this sense, our careful assessment should have consolidated our orthologous sequence dataset.

### Batoidea–Selachimorpha split in the late Carboniferious period

The earliest fossils assigned to Chondrichthyes are assigned to the Silurian (444–416 Ma), and later chondrichthyan fossils become more widespread in the Devonian (416–359 Ma) [5]. Based on the fossil evidence, Holocephali and Elasmobranchii are estimated to have diverged 410 Ma [5]. For our estimation of the Holocephali-Elasmobranchii split, likewise with Inoue et al. [15], we adopted the time constraint for the split between Osteichthyes and Chondrichthyes (Table S2) based on Benton et al. [35], to stabilize the adjacent younger node of the Holocephali-Elasmobranchii split. Thus, our analysis employing nuclear sequence data yielded a similar divergence time estimate of 421 Ma (410–441Ma).

Moreover, for the estimation of the Batoidea-Selachimorpha divergence time, a study by Klug et al. attracted our attention. In this study, the †Synechodontiformes were identified as monophyletic group, sister to all extant sharks [19]. Klug *et* *al.* discuss that this assignment makes it necessary to enlarge the concept of neoselachian systematics to include this completely extinct group, which is considered to represent stem-group neoselachians [19]. The earliest fossils of †Synechodontiformes were assigned to the Early Permian (295 Ma) [36], and Klug *et* *al.* concluded that the origin of neoselachians can be as young as the Late Permian about 250 Ma [19]. Applying this above-mentioned record of 250 Ma for the Batoidea-Selachimorpha divergence as a minimum time constraint, presuming Batoidea as sister group to Selachimorpha, our calculation resulted in a mean divergence time of 306 Ma (252–387 Ma). In comparison with Heinicke et al. indicating a much older divergence of 393 Ma [20], our estimate would need to assume a shorter ghost range (306–250 Ma) based on the fossil records for early divergences within Neoselachians.

### Possible sources of further improvement

It could be practical to discuss what might higher the resolution of the study. One simplistic idea is to increase the sequence information. At the moment, there is no truly genome-wide resource for any chondrichthyan species – the *C. milii* genome was only highly partially sequenced, and the resulting assembly does not cover many universal genes [37]. It can be augmented with deep transcriptomic sequencing. Apart from the amount of original sources, phylogenetic marker gene selection can also result in a remarkable difference. Our orthology assessment, primarily based on the Blast bit scores and secondarily on ML tree inferences, played a crucial role in removing any noisy data caused by possibly non-orthologous gene set for divergence time estimation. However, our rigorous criteria led to a relatively small number of phylogenetic marker genes (Table 1). While being aware of its risk to include possible cases with hidden paralogy, relaxing the selection criteria regarding orthology may result in a large increase in the number of genes in divergence time estimation. Another possible source of improvement lies in taxon sampling. Flexible choice of species in ingroup or outgroup may lead to reservation of more sites in the alignment used in divergence time estimation.

On the other hand, inclusion of more fossil records could also largely improve the results. In this study, divergence time constraints based on fossil records were narrow enough for non-chondrichthyan lineages. Apparently, fossil records in the chondrichthyan lineages are currently scarce and remain to be augmented by future effort.

### Decreased molecular evolutionary rates in chondrichthyan lineages

The decreased rate of molecular evolution for a number of chondrichthyan species is already suggested since some chondrichthyan species have been subjected to various molecular analyses [21]–[25]. Mulley *et* *al.* suggested that the genomes of cartilaginous fish generally are more highly conserved than those of tetrapods or teleost fish, whereas for instance the investigation by Ravi *et* *al.* showed that teleosts show higher rate of chromosomal rearrangements and that protein-coding sequences in teleost fish genomes are evolving faster than in mammals [22], [38]. Based on the slow evolution observed in the individual genes of our study (Table 3), we support the hypothesis by Mulley *et* *al.* that the entire genomes of chondrichthyans may be evolving more slowly. On the other hand, the loss of the Hox C cluster was implicated in the elasmobranch lineage [39], and this is a remarkable drastic change that has never been observed in any other jawed vertebrate lineage. This contrast, seen between different features of the genomes, demands a caution in discussing rates of molecular evolution.

A question whether rate of morphological evolution is associated with that of molecular evolution has been repeatedly discussed regarding so-called ‘living fossils’, such as tuatara [40], [41] and coelacanth [42], [43]. To date, embryonic development was investigated in detail for some chondrichthyan species including the small spotted catshark *Scyliorhinus canicula* [44], the chimaera *Callorhinchus milii* [45] and the clearnose skate, *Raja eglanteria* [46]. Including more representatives of currently missing lineages, it is expected to get thorough and quantitative framework of morphological evolution. Our analysis of molecular evolutionary rate focusing on Hox A cluster resulted in at least 2.4-fold decrease in the chondrichthyan lineage in comparison to the tetrapod lineage, which remains to be confirmed at a genomic scale.

### Conclusions

Large-scale data of nuclear gene sequences for chondrichthyan species were collected. We employed the amino acid sequence alignment with 2973 unambiguously aligned amino acid sites of 20 protein-coding genes in Bayesian-based divergence time analyses. Assuming an origin of sharks in the late Permian no less than 250 Ma and the batoids as sister clade to sharks within Neoselachii, result in a mean divergence time of 306 Ma for the Selachimorpha-Batoidea split.

Moreover, our analysis revealed a lower rate of molecular evolution for chondrichthyan lineages by a factor of at least 2.4.

## Methods

### Bioinformatic analysis pipeline

The procedure for this step is outlined in Figure 2. Expressed sequence tags (ESTs) of four elasmobranch species, *Squalus acanthias* [47], *Leucoraja erinacea* [47], *Chiloscyllium plagiosum* and *Torpedo californica* were downloaded from NCBI and assembled with the program Phrap [48], [49] with the default parameters. All resulting contigs and singletons were comprehensively translated into peptide sequences with all possible six open reading frames. The genome sequences of *Callorhinchus milii* were subjected to *ab initio* gene prediction with the program GenScan [50]. Using each peptide sequence of the predicted *C. milii* genes plus all annotated peptide sequences for chimaeras as a query, Blastp searches were performed towards three peptide sequence databases for 1) sharks, 2) rays/skates and 3) human (as outgroup). The human sequences were retrieved from NCBI RefSeq. The shark and ray/skate sequence collections included annotated peptide sequences retrieved from NCBI GenBank. As the preliminary orthology assessment step, the bit scores of the Blastp searches [51] were evaluated so that *C. milii* query sequences whose bit score in the search towards human (*S*_co_) is smaller than those in the search towards both sharks and rays/skates (*S*_cs_ and *S*_cr_). As a result, 203 genes were passed onto downstream analyses.

### Phylogenetic analysis and taxon sampling

For each of the 203 selected candidate genes which passed the preliminary orthology assessment step (Figure 2), homologous sequences of bilaterians to each *C. milii* sequences selected in the preliminary orthology assessment step were retrieved from NCBI GenBank and Ensembl to build molecular phylogenetic trees. First a multiple alignment of amino acid sequences was constructed using the alignment editor XCed in which the alignment algorithm MAFFT is implemented [52]. The 5′ and 3′ ends of the alignment that contained large stretches of missing data, compared with the chondrichthyan species, were truncated to include the most efficient number of unambiguously aligned sites and resulted in alignments between 98 aa [for *M-phase phosphoprotein 10* (*MPP10*) gene] and 381 aa [for *phosphoglycerate kinase 1* (*PGK1*) gene].

Preliminary neighbor joining (NJ) trees [53] were inferred on XCed. The candidate genes in which a rough orthology could be confirmed were further analyzed in more detail, by inferring their trees by the maximum-likelihood (ML) method [54] using PHYML version 2.4.4 [55], assuming the JTT+I+Γ_4_ model. Generally, to avoid wrong assignment of orthology we started with a more extensive dataset including a large number of bilaterian sequences. In several rounds of NJ and ML analyses, we basically retained orthologs of 12 core species for the final data set with few exceptions (Table S1). This data set included four representatives of tetrapods (*Homo sapiens*, *Monodelphis domestica*, *Gallus gallus* and *Xenopus tropicalis*), three teleost fish species (*Oryzias latipes*, *Takifugu rubripes* and *Danio rerio*) and three selected chondrichthyans, namely one representative of each lineage (Holocephali, Batoidea and Selachimorpha). *Ciona intestinalis* and *Drosophila melanogaster* were used as outgroup.

In each step of refinement, we needed to be careful in use of these nuclear genes not to overlook ‘hidden paralogy’ [56], [57]. For instance, secondary losses or delayed identifications of gene family members after the 2R-WGDs [58] could lead to a confusing pattern, in which paralogous sequences appear to be orthologous [59]. For candidate genes in which a pattern of the teleost-specific genome duplication (TSGD) was observed [60], the more divergent subtype of each teleost fish species was excluded for further analyses. If the orthology of a candidate could not be confirmed, these sequences were not incorporated in the following phylogenetic analyses. Generally, resultant tree topologies were assessed in light of the tree topology supported by Kikugawa et al. [1].

For all groups in which an orthology could be confirmed but differences of relationships between groups emerged, we finally calculated the log-likelihoods (log*L*) of two trees. First, applying the currently accepted species phylogeny and second, assuming the phylogeny maintained in the ML analysis, using TREE-PUZZLE version 4.2 [61]. We compared the determined likelihoods of the two trees and considered their difference (*Δ*log*L)*. If the standard error (SE) of the second-best calculated likelihood was larger than the *Δ*log*L* of the two trees, these provided candidate genes were included in further analysis for divergence time estimation (*Δ*log*L*/SE<1, accepted; *Δ*log*L*/SE>1, rejected).

### Divergence time inference

For the divergence time analysis, a Bayesian-based method implemented in the MCMCTREE program in the PAML 4.4 package [62] was used, implying a relaxed molecular clock that take into account rate variation across lineages (clock  = 2) [63]. The unambiguously assigned alignment sites of each candidate were concatenated to one input file. The assumed tree topology, accepted for all single genes in the likelihood analysis, was applied and each node outside the chondrichthyan lineage, except Chordata, was constrained by a soft minimum and maximum calibration point (Table S2) based on the fossil record [35]. For the Selachimorpha/Batoidea split, in one analysis, a calibration point of 190 Ma was adopted according to a previous study [15] and in another, we adopted a lower constraint of 250 Ma based on a recently reported fossil record [19]. Inside the chondrichthyan lineage only a lower hard time constraint was applied. Using CODEML implemented in the PAML package, the substitution rate and the gamma shape parameter alpha was estimated using the JTT model [64]. Two priors were set for the final MCMCTREE analysis, the overall substitution rate (rgene_gamma) at G (1, 5.2) and the rate-drift parameter (sigma2_gamma) at G (1, 5.6) for our dataset containing 2973 amino acids (20 nuclear genes).

One time unit was used as 100 million years, because the node ages should fall between 0.01 and 10 [62]. Markov chain Monte Carlo (MCMC) approximation with a burn-in period of 50,000 cycles was obtained, and every 20 cycles were taken to create a total of 200,000 samples. Two replicates with different random seed numbers were performed to work out possible failure of the Markov chains to converge to their stationary distribution [15]. After completion of the analysis the output was plotted in TRACER 1.5 (available at http://beast.bio.ed.ac.uk/Tracer) to re-examine their convergence and to obtain the marginal densities of the calculated mean (Figure 4).

### Quantification of evolutionary rates

To infer evolutionary rates of three chondrichthyan representatives [*Scyliorhinus canicula* (*Sc*), *Leucoraja erinacea* (*Le*) and *Heterodontus francisci* (*Hf*)] in comparison to tetrapods [human (*Hs*), chicken (*Gg*), *Anolis carolinensis* (*Ac*) and *Xenopus tropicalis* (*Xt*)] we downloaded all available amino acid sequences of Hox A protein-coding genes from GenBank [65] or Ensembl [66] for the above-mentioned species as well as for the elephant shark [*Callorhinchus milii* (*Cm*)] (accession numbers: Table S3). All available Hox A genes (*HoxA1-A7*, *A9-A11* and *A13*) were concatenated into one sequence file for each species and we created six amino acid alignment datasets using the alignment editor XCed. The numbers of unambiguously aligned amino acid sites in Hox A protein-coding alignments are found in Table 3. These sites were extracted into a new input file and the distances (number of substitutions per site) between each pair of species were calculated running CODEML of the PAML 4.4 package [62], applying the JTT model [65]. A loose bound for the root was set at 420 Ma (in dataset 1–3), and at 450 Ma (in dataset 4–6). To further calculate the distance of each ingroup species (A, B) to a hypothetical ancestor (O) a relative rate test was performed with an outgroup (C) as below.
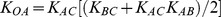


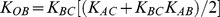



To infer absolute rates of evolution, the calculated distances (*K*_OA_ and *K*_OB_) were divided through our estimated divergence time (306 Ma for shark-ray, dataset 2 and 3), as well as previously estimated divergence times (203 Ma [15] for *Hf*-*Sc* in dataset 1, 312 Ma [35] for *Hs*-*Gg* and *Hs*-*Ac* in dataset 4 and 5, and 330 Ma [35] for *Hs*-*Xt*, respectively) and thus we performed a quantification of divergence rates by pairwise comparison.

In a supplementary investigation, we employed the complete Hox A cluster nucleotide sequences to calculate pairwise distances and evolutionary rates of species described above. We downloaded sequence data of whole Hox A clusters from Ensembl [66]: *Hs* (GhRC37, chromosome 7, base position 27128722 to 27250000), *Gg* (WASHUC2.1, chromosome 2, base position 32508052 to 32636817), *Ac* (AnoCar2.0, Scaffold GL343275.1, base position 1364049 to 1613271, reverse complement (r.c.)) and *Xenopus tropicalis* (assembly 4.2 by the Joint Genome Institute, Scaffold GL172692.1, base position 1377582 to 1529191, r.c.) and GenBank [65]: *Sc* (FQ032658.1, r.c.), *Le* (FJ944024.1, r.c.), *Hf* (AF224262.1, r.c.; AF479755.1, r.c.) and *Cm* (FJ824598.1). All Hox A cluster sequences were aligned using mVista [67]. The multiple alignment was transferred to MEGA 5 and a complete deletion of gaps and missing data was set. This condition finally resulted in 8081 nucleotide sites, for which pairwise distances were computed [68] with the *p*-distance method.

## Supporting Information

Table S1 **List of species included in this analysis.**
(PDF)

Table S2 **List of time constraints.** Upper (U) and lower (L) time constraints in million years from present (Ma) applied for nodes in estimating divergence times in the chondrichthyan lineage.
(PDF)

Table S3 **Accession numbers of Hox A proteins employed in the evolutionary rate analysis.** The sequences with the given accession numbers were retrieved from the NCBI or Ensembl database.
(PDF)
