# Supplementary material for: Revealing Less Derived Nature of Cartilaginous Fish Genomes with Their Evolutionary Time Scale Inferred with Nuclear Genes
Source: PLoS One. 2013 Jun 25;8(6):e66400. doi: 10.1371/journal.pone.0066400 (PMC3692497; doi:10.1371/journal.pone.0066400)
Supplement: Table S1 — List of species included in this analysis. (PDF) [file pone.0066400.s001.pdf]

Table S1: List of species used for divergence time analysis

Class or subclass<br>family	Species
Insecta	
Drosophilidae	*Drosophila melanogaster*
	Drosophila simulans
Ascidiacea	
Cionidae	*Ciona intestinalis*
Actinopterygii	
Tetraodontidae	*Takifugu rubripes*
Adrianichthyidae	*Oryzias latipes*
Cyprinidae	*Danio rerio*
Sarcopterygii	
Pipidae	*Xenopus tropicalis*
Phasianidae	*Gallus gallus*
Didelphidae	*Monodelphis domestica*
Hominidae	*Homo sapiens*
Elasmobranchii	
Squalidae	*Squalus acanthias*
Hemiscylliidae	*Chiloscyllium plagiosum*
Rajidae	*Leucoraja erinacea*
Torpedinidae	*Torpedo californica*
Holocephali	
Callorhinchidae	*Callorhinchus callorynchus*
	Callorhinchus milii
