# Supplementary material for: Revealing Less Derived Nature of Cartilaginous Fish Genomes with Their Evolutionary Time Scale Inferred with Nuclear Genes
Source: PLoS One. 2013 Jun 25;8(6):e66400. doi: 10.1371/journal.pone.0066400 (PMC3692497; doi:10.1371/journal.pone.0066400)
Supplement: Table S2 — List of time constraints. Upper (U) and lower (L) time constraints in million years from present (Ma) applied for nodes in estimating divergence times in the chondrichthyan lineage. (PDF) [file pone.0066400.s002.pdf]

Table S2: List of time constraints

Node #	Time (Ma)		Divergence
	U	L	
1	581	531	Protostomia/Deuterostomia
2	-	518	Gnathostomata/Tunicata
3	463	421	Osteichthyes/Chondrichthyes
4	421	416	Sarcopterygii/Actinopterygii
5	351	330	Amphibia/Amniota
6	331	312	Birds/Mammals
7	171	124	Marsupials/Placentals
8	163	149	Otocephala/Euteleostei
9	151	96	Tetraodontiformes/Beloniformes
10	-	410	Holocephali/Elasmobranchii
11 - I	-	250	Selachimorpha/Batoidea
11 - II	-	190	

Maximum (Upper, U) and minimum (Lower, L) time constraints in million years from present (Ma) applied for nodes in estimating divergence times in the chondrichthyan lineage.
