# Supplementary material for: Revealing Less Derived Nature of Cartilaginous Fish Genomes with Their Evolutionary Time Scale Inferred with Nuclear Genes
Source: PLoS One. 2013 Jun 25;8(6):e66400. doi: 10.1371/journal.pone.0066400 (PMC3692497; doi:10.1371/journal.pone.0066400)
Supplement: Table S3 — Accession numbers of Hox A proteins employed in the evolutionary rate analysis. The sequences with the given accession numbers were retrieved from the NCBI or Ensembl database. (PDF) [file pone.0066400.s003.pdf]

Table S3: Accession numbers of Hox protein sequences employed in evolutionary rate analysis

Gene	*Homo sapiens*	*Anolis carolinensis*	*Gallus gallus*	*Xenopus tropicalis*	*Callorhinchus milii*	*Scyliorhinus canicula*	*Heterodontus francisci*	*Leucoraja erinacea*
HoxA1	AAB35423	ENSACAP 00000010763	AAO37643 (partial)	ENSXETP 00000001600	ACU32554	CBL59343	AAF44639	ACT65756
HoxA2	NP_006726	ENSACAP 00000010237	NP_990481	ENSXETP 00000001601	ACU32553	CBL59342	AAF44640	ACT65755
HoxA3	AAH15180	ENSACAP 00000010220	NP_989879	ENSXETP 00000001602	ACU32552	CBL59341	AAF44641	ACT65754
HoxA4	NP_002132	ENSACAP 00000010189	CAA36896	ENSXETP 00000057392	ACU32551	CBL59340	AAF44642	ACT65753
HoxA5	CAG47073	ENSACAP 00000010179	AAT90845	ENSXETP 00000001604	ACU32550	CBL59339	AAF44643	ACT65752
HoxA6	NP_076919	-	AAQ81318	ENSXETP 00000047590	ACU32549	CBL59338	AAF44644	ACT65751
HoxA7	CAA06713	ENSACAP 00000010136	NP_989926	ENSXETP 00000001606	ACU32548	CBL59337	AAF44645	ACT65750
HoxA9	NP_689952	ENSACAP 00000007125	Q98924 (partial)	ENSXETP 00000001611	ACU32547	CBL59336	AAF44646	ACT65749
HoxA10	NP_061824	ENSACAP 00000010110	ENSGALP 00000018009	ENSXETP 00000001618	ACU32546	CBL59335	AAF44647	ACT65748
HoxA11	NP_005514	ENSACAP 00000010103 (partial)	NP_989950	ENSXETP 00000001623	ACU32545	CBL59334	AAM18672	ACT65747
HoxA13	AAC50993	ENSACAP 00000010093	NP_989470	ENSXETP 00000001626	ACU32544	CBL59333	AAM18671	ACT65746

Note: Accession numbers are obtained from the NCBI or Ensembl database.
